# Response to “Addressing biases and limitations in feature attribution for circRNA modification profiling”

**DOI:** 10.1093/bib/bbag161

**Published:** 2026-04-08

**Authors:** Jiayi Li, Shenglun Chen, Zhixing Wu, Haozhe Wang, Rong Xia, Jia Meng, Yuxin Zhang

**Affiliations:** Department of Biosciences and Bioinformatics, School of Science, Xi'an Jiaotong-Liverpool University, 111 Ren'ai Road, Suzhou Industrial Park, Suzhou, Jiangsu 215123, China; Center for Intelligent RNA Therapeutics, Suzhou Key Laboratory of Cancer Biology and Chronic Disease, Xi'an Jiaotong-Liverpool University, 111 Ren'ai Road, Suzhou Industrial Park, Suzhou, Jiangsu 215123, China; Institute of Systems, Molecular and Integrative Biology, University of Liverpool, Biosciences Building, Crown Street, Liverpool L69 7ZB, United Kingdom; Department of Biosciences and Bioinformatics, School of Science, Xi'an Jiaotong-Liverpool University, 111 Ren'ai Road, Suzhou Industrial Park, Suzhou, Jiangsu 215123, China; Center for Intelligent RNA Therapeutics, Suzhou Key Laboratory of Cancer Biology and Chronic Disease, Xi'an Jiaotong-Liverpool University, 111 Ren'ai Road, Suzhou Industrial Park, Suzhou, Jiangsu 215123, China; Institute of Infection, Veterinary and Ecological Sciences, University of Liverpool, Leahurst Campus, Neston, Brownlow Hill, Wirral CH64 7TE, United Kingdom; Department of Biosciences and Bioinformatics, School of Science, Xi'an Jiaotong-Liverpool University, 111 Ren'ai Road, Suzhou Industrial Park, Suzhou, Jiangsu 215123, China; Center for Intelligent RNA Therapeutics, Suzhou Key Laboratory of Cancer Biology and Chronic Disease, Xi'an Jiaotong-Liverpool University, 111 Ren'ai Road, Suzhou Industrial Park, Suzhou, Jiangsu 215123, China; The School of AI and Advanced Computing, XJTLU Entrepreneur College (Taicang), Xi'an Jiaotong-Liverpool University, 5th Floor, Building D, No. 111 Road, Taicang Avenue, Taicang, Suzhou, Jiangsu 215400, China; Department of Computer Science, University of Liverpool, Ashton Building, Liverpool, Merseyside L69 3DR, United Kingdom; Department of Biosciences and Bioinformatics, School of Science, Xi'an Jiaotong-Liverpool University, 111 Ren'ai Road, Suzhou Industrial Park, Suzhou, Jiangsu 215123, China; Center for Intelligent RNA Therapeutics, Suzhou Key Laboratory of Cancer Biology and Chronic Disease, Xi'an Jiaotong-Liverpool University, 111 Ren'ai Road, Suzhou Industrial Park, Suzhou, Jiangsu 215123, China; Department of Computer Science, University of Liverpool, Ashton Building, Liverpool, Merseyside L69 3DR, United Kingdom; Department of Biosciences and Bioinformatics, School of Science, Xi'an Jiaotong-Liverpool University, 111 Ren'ai Road, Suzhou Industrial Park, Suzhou, Jiangsu 215123, China; Center for Intelligent RNA Therapeutics, Suzhou Key Laboratory of Cancer Biology and Chronic Disease, Xi'an Jiaotong-Liverpool University, 111 Ren'ai Road, Suzhou Industrial Park, Suzhou, Jiangsu 215123, China; The School of AI and Advanced Computing, XJTLU Entrepreneur College (Taicang), Xi'an Jiaotong-Liverpool University, 5th Floor, Building D, No. 111 Road, Taicang Avenue, Taicang, Suzhou, Jiangsu 215400, China; Department of Computer Science, University of Liverpool, Ashton Building, Liverpool, Merseyside L69 3DR, United Kingdom; Department of Biosciences and Bioinformatics, School of Science, Xi'an Jiaotong-Liverpool University, 111 Ren'ai Road, Suzhou Industrial Park, Suzhou, Jiangsu 215123, China; Center for Intelligent RNA Therapeutics, Suzhou Key Laboratory of Cancer Biology and Chronic Disease, Xi'an Jiaotong-Liverpool University, 111 Ren'ai Road, Suzhou Industrial Park, Suzhou, Jiangsu 215123, China; Institute of Systems, Molecular and Integrative Biology, University of Liverpool, Biosciences Building, Crown Street, Liverpool L69 7ZB, United Kingdom; Department of Biosciences and Bioinformatics, School of Science, Xi'an Jiaotong-Liverpool University, 111 Ren'ai Road, Suzhou Industrial Park, Suzhou, Jiangsu 215123, China; Center for Intelligent RNA Therapeutics, Suzhou Key Laboratory of Cancer Biology and Chronic Disease, Xi'an Jiaotong-Liverpool University, 111 Ren'ai Road, Suzhou Industrial Park, Suzhou, Jiangsu 215123, China; Institute of Systems, Molecular and Integrative Biology, University of Liverpool, Biosciences Building, Crown Street, Liverpool L69 7ZB, United Kingdom; State Key Laboratory of Epigenetic Regulation and Intervention, Institute of Biophysics, Chinese Academy of Sciences, No. 15 Datun Road, Chaoyang District, Beijing 100101, China

**Keywords:** circRNA, feature attribution, model interpretability

## Abstract

This response addresses the comments raised by Souichi Oka and colleagues in their Letter to the Editor titled “Addressing biases and limitations in feature attribution for circRNA modification profiling.” We clarify that two independent XGBoost models were used for distinct purposes in our analysis: one for predicting RNA modification events from nanopore-derived signal features and another for feature attribution using genome-derived sequence features extracted through the m6AlogisticModel framework. We further note that Shapley Additive Explanations (SHAP) was employed as an exploratory interpretability tool rather than as definitive evidence of causal biological mechanisms. We appreciate the constructive methodological suggestions provided and acknowledge that integrating complementary analytical strategies may further enhance the robustness of computational studies of circRNA modifications.

Dear Editor,

We thank Souichi Oka and colleagues for their thoughtful comments on our article, CircRM: profiling circular RNA modifications from nanopore direct RNA sequencing. Their discussion raises several important issues concerning the interpretation of feature importance and the methodological limitations of machine-learning–based analyses in epitranscriptomic studies. We appreciate the opportunity to clarify several aspects of our study.

First, we would like to clarify the modeling framework used in our analysis. In our study, two distinct XGBoost models were constructed for different analytical purposes. The first model, which incorporated nanopore signal-derived features such as dwell time and other signal statistics, was developed solely for the prediction of RNA modification events. In contrast, the feature attribution analysis reported in our study was performed using a second, independently trained model. For this analysis, genome-derived sequence features were extracted using the feature representation strategy implemented in m6AlogisticModel [1], after which a separate XGBoost model was trained based on these features. Importantly, this second model is independent of the prediction model based on nanopore signal features. Therefore, the SHAP-based interpretation presented in our study reflects the behavior of the sequence-feature model rather than the signal-based prediction model.

Second, the use of SHAP for model interpretation represents a widely adopted approach in machine learning and bioinformatics. SHAP provides a theoretically grounded framework for estimating feature contributions based on cooperative game theory and has been widely used to interpret complex predictive models across diverse biological applications [2, 3]. While we acknowledge that no attribution method is without limitations, SHAP remains a commonly used tool for examining how predictive features contribute to model outputs. In our study, SHAP analysis was used primarily as an exploratory approach to identify features that may be associated with the model’s predictions, rather than as definitive evidence of causal biological mechanisms.

Third, we appreciate the authors’ constructive suggestions regarding potential methodological improvements. In particular, approaches such as highly variable feature selection, feature agglomeration, and complementary non-parametric statistical analyses may provide additional perspectives for evaluating feature relevance in complex biological datasets. We agree that incorporating multiple analytical perspectives can strengthen the robustness of computational inference. These suggestions are valuable and will be considered in future work aimed at further improving the analytical framework for circRNA modification profiling.

In summary, we thank the authors for their constructive comments and for highlighting important considerations regarding model interpretability. We hope that the clarifications provided here help to better explain the analytical design of our study. Continued methodological discussion will undoubtedly contribute to improving computational approaches for studying RNA modifications.

**Key Points**

- CircRM employs two independent XGBoost models for prediction and interpretability analyses.
- SHAP was applied as an exploratory interpretability tool to examine feature contributions to model predictions rather than to establish causal biological mechanisms.
- The methodological suggestions proposed in the Letter are appreciated and may help further improve the robustness of computational analyses for circRNA modification profiling.

## Data Availability

There are no new data associated with this article.
